# Application of Machine Learning for Drug–Target Interaction Prediction

**DOI:** 10.3389/fgene.2021.680117

**Published:** 2021-06-21

**Authors:** Lei Xu, Xiaoqing Ru, Rong Song

**Affiliations:** ^1^School of Electronic and Communication Engineering, Shenzhen Polytechnic, Shenzhen, China; ^2^Department of Computer Science, University of Tsukuba, Tsukuba, Japan

**Keywords:** machine learning, drug–target interactions, data, features, task algorithms, drug development

## Abstract

Exploring drug–target interactions by biomedical experiments requires a lot of human, financial, and material resources. To save time and cost to meet the needs of the present generation, machine learning methods have been introduced into the prediction of drug–target interactions. The large amount of available drug and target data in existing databases, the evolving and innovative computer technologies, and the inherent characteristics of various types of machine learning have made machine learning techniques the mainstream method for drug–target interaction prediction research. In this review, details of the specific applications of machine learning in drug–target interaction prediction are summarized, the characteristics of each algorithm are analyzed, and the issues that need to be further addressed and explored for future research are discussed. The aim of this review is to provide a sound basis for the construction of high-performance models.

## Introduction

Tens of thousands of known diseases threatening human health, and new ones are being added every year. They include emerging diseases (e.g., the currently prevalent COVID-19) and diseases that have plagued the public for many years and have no cure so far (e.g., Parkinson’s disease and Alzheimer’s disease) (Xu et al., 2018a, 2019). Rapidly and accurately discovering drugs that can effectively treat diseases is very important for the development of society. Long cycle and high cost are common phenomena in current drug development, but these fail to guarantee a high success rate. Many steps are required from drug development to final marketing, including drug discovery, preclinical and clinical trials, and marketing approval (Srivastava et al., 2019; Li Z. et al., 2020). The overall success rate of drug discovery and preclinical studies, which are part of the laboratory development phase, is approximately 0.05–0.1%, and less than 1% of the candidate compounds are likely to have the expected effect and proceed to the clinical trial phase. Investigating drug–target interactions is an important step in the drug discovery process and can improve the success rate of new drug discovery (Chen et al., 2019; Huang et al., 2020; Zeng et al., 2020b). These not only signal the need to expend significant resources to find and test candidate compounds one by one during the drug development phase to confirm that they meet expectations, but also demonstrate the importance of drug–target interaction prediction in the overall drug development process. Supplementally, an obvious drawback of biomedical experiment is that it does not allow for rapidly finding and solving problems, which can be detrimental to the treatment of emerging and highly infectious diseases. Therefore, machine learning methods have been introduced into the prediction of drug–target interactions.

Machine learning, a computer technology for data analysis designed to build predictive models using datasets, has become an important means of modern biological research (Xu et al., 2018b; Yang et al., 2018; Liu et al., 2019, 2020; Tang et al., 2020; Zeng et al., 2020a). It has become a mainstream technique for analyzing and solving problems involved in drug–target interaction prediction studies (Cai et al., 2018; Stephenson et al., 2019; Zeng et al., 2019; Fu et al., 2020; Wang J. et al., 2020).

## Three Factors

The existing data background, powerful toolkits, and current status and requirements have promoted machine learning to become the mainstream method of drug–target interaction prediction.

(1) Existing databases. With the emergence of sequencing technology, high-throughput technology and computer-aided drug design method, a large number of proteins have been sequenced and many compounds have been synthesized. On the basis of existing related works and accumulated experience, relevant data has been organized and various databases have been constructed. Most of the data in these databases are publicly available and free to download, which provides a good data foundation for solving drug–target interaction prediction problems by machine learning. Researchers can collect datasets from databases that cover different information according to their needs (Zheng et al., 2019, 2020). Some representative databases are briefly described here.

UniProt database^1^ : UniProt is supported by many institutions, and is the most informative and comprehensive protein database (Consortium, 2015). It consists of five sub-databases: Swiss-Prot, TrEMBL, UniRef, UniParc, and Proteomes. Each sub-database has its own unique function. For example, Swiss-Prot is a high-quality, manually annotated, non-redundant database, in which protein annotations are derived mainly from the literature or E-value verification calculation analysis results. Proteomes is a database that provides proteomic information for species with fully sequenced genomes.

PubChem database^2^ : PubChem is an open chemistry database that collects information including chemical structures, identifiers, physicochemical properties, and biological activities of chemical molecules (Kim et al., 2016, 2021). It is the world’s largest database with free access to chemical information, and currently covers 109 million compounds. PubChem has become an important chemical information resource for scientists, students, and the public.

DrugBank database^3^ : As a bioinformatics and cheminformatics resource, DrugBank combines detailed drug data (i.e., chemical, pharmacological, and pharmaceutical) with comprehensive target information (i.e., sequence, structure, and pathway) (Wishart et al., 2018). The latest DrugBank release (version 5.1.8.) contains 14,443 drug molecules and 5,244 non-redundant protein sequences associated with these drugs. The database describes not only clinical information on drugs, namely drug side effects and drug–drug interactions, but also contains molecular-level data, such as chemical structures of drugs and proteins targeted by drugs (Wishart et al., 2008). One significant function of DrugBank is that it supports comprehensive and complex searches, so it is used widely by the pharmaceutical industry, medicinal chemists, pharmacists, physicians, students, and the general public.

KEGG database^4^ : KEGG was established in 1995 by the Kanehisa Laboratories at the Bioinformatics Center, Kyoto University, Japan, and is now one of the most commonly used international bioinformatics databases (Kanehisa and Goto, 2000). KEGG is a database used to understand the high-level functions and practicability of biological systems from molecular-level information (Li H. et al., 2020; Wang et al., 2021a) (especially large-scale molecular datasets generated by genome sequencing and other high-throughput techniques), of which the data information can be roughly classified into four major categories: system information, genetic information, chemical information, and medical information.

BindingDB database^5^ : BindingDB is a publicly available, web-accessible database for measuring binding affinity, focusing on the interactions between proteins considered to be drug targets and drug-like small molecules (Liu et al., 2007). BindingDB currently contains 2,114,159 binding data between 8,202 protein targets and 928,022 small molecules.

(2) Powerful toolkits and web servers. Bioinformatics and cheminformatics are emerging interdisciplinary fields that use computers to solve biological and chemical problems. Many toolkits and web servers have been developed (Zuo et al., 2017; Zou et al., 2019; Lin et al., 2020; Pang and Liu, 2020; Shao et al., 2021), which can help to solve problems in drug–target interaction prediction.

STITCH^6^ : STITCH not only includes experimentally validated drug–target interaction data, but also integrates predicted drug–target relationships (Kuhn et al., 2007). This website can clearly depict the protein–protein interactions, protein–compound interactions, and the strength of the interactions.

SwissTargetPrediction^7^ : SwissTargetPrediction can estimate the most likely macromolecule to be targeted by a biologically active small molecule and count the percentage of each target type targeted by the small molecule (Gfeller et al., 2014).

RDkit^8^ : RDkit is a powerful python toolkit for chemical information, which has functions such as acquiring molecule information from multiple formats, obtaining information about atoms, bonds, and rings in molecules, generating molecular descriptors and molecular fingerprints of compounds, and calculating similarities of compound structures (Landrum, 2013).

OpenChem^9^ : OpenChem is a pytorch-based deep learning toolkit for computational chemistry and drug design, which contains Feature2Label, Smiles2Label, Graph2Label, SiameseModel, GenerativeRNN, and MolecularRNN (Korshunova et al., 2021). Users can train predictive models for classification, regression, and multi-task problems, and develop generative models for generating novel molecules with optimized properties. Its goal is to make deep learning an easy-to-use tool for researchers in computational chemistry and drug design.

iFeature^10^ : iFeature is a python toolkit that can compute various structural and physicochemical property descriptors from protein and peptide sequences. iFeature can compute and extract comprehensive spectra for 18 major sequence coding schemes, including 53 different types of feature descriptors. In addition, iFeature integrates 12 different types of commonly used feature clustering, selection, and dimensionality reduction algorithms (Chen et al., 2018).

Pse-in-one^11^ : Pse-in-one is a python toolkit that generates all possible pseudo-components of DNA, RNA, and protein sequences. It covers a total of 28 different patterns, 14 for DNA sequences, 6 for RNA sequences, and 8 for protein sequences (Liu et al., 2015, 2017). This toolkit is widely and increasingly used by researchers to tackle various problems in computational biology, and a more specific and detailed version BioSeq-Analysis (Liu, 2019) has recently been released.

(3) Current status and requirements. With the development of high-throughput technologies, many compounds and proteins have been mined. The human genome contains more than 20,000 genes, and approximately 80% of them can encode one or more proteins. Only a small number of proteins have been identified as pharmacologically active and are targets for currently approved drugs. The pharmacological functions of most proteins remain to be demonstrated. This is also true for most compounds. For example, there are currently 111 million compounds in the PubChem database, but proteins that could interact with many of these compounds are unknown. In addition, it is obvious that the traditional approach of wet experiments is not feasible for some emerging, highly infectious and destructive new pathogens, such as the SARS, H7N9, Ebola, Mers, and COVID-19 viruses (Cheng et al., 2021). Considering the huge amounts of available data and large numbers of diseases that cause serious social health risks, using computational chemistry-related theories and computer simulation methods to computationally predict drug–target interaction can effectively improve efficiency. Machine learning-based methods have become effective ways to compensate for the shortcomings of traditional biochemical experimental methods.

## Applications

**FIGURE 1:**
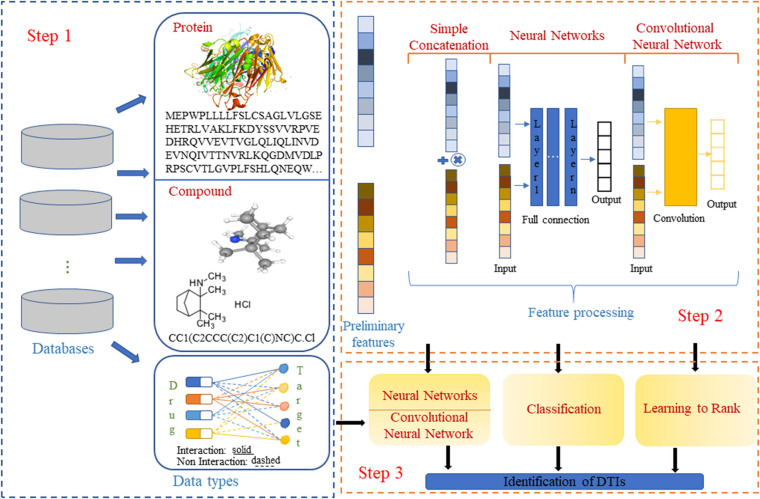
Steps for predicting drug-target interactions. The two- and three-dimensional structure diagrams of the drug are from PubChem.

The current drug–target interaction prediction procedures are shown in Figure 1. Existing studies on drug–target interaction prediction have shown that using different calculation or optimization methods in the steps of data set acquisition, feature extraction and processing, and task algorithm selection can build models with good performance.

(1) Dataset acquisition. Redundant data, unbalanced categories, and unrepresentative samples can lead to long experimental cycles, as well as inaccurate and biased experimental results. Different data acquisition methods have been used to avoid or reduce the impact of these problems on model construction. For example, Wang et al. (2010) collected negative examples by random selection to solve the data imbalance problem. Wang et al. (2018) also used random selection to extract negative examples, and this operation was performed five times to reduce the impact of the unverified negative samples. Pdti-EssB (Mahmud et al., 2020) used random under-sampling and under-sampling clustering to address the data imbalance problem.

Currently, most target molecules are proteins, of which four protein families [kinases, G protein-coupled receptors (GPCRs), ion channels, and nuclear receptors] account for 44% of the target molecules, and 70% of the currently developed drugs are targeted to these four protein families. Datasets established by Yamanishi et al. (2008), which contain the interactions between these four proteins and drugs, have been widely used (Öztürk et al., 2018; Mahmud et al., 2020). The relevant data can be downloaded from http://web.kuicr.kyoto-u.ac.jp/supp/yoshi/drugtarget/. Most of the computational approaches based on these datasets have focused on binary classification, that is, they only explore whether a drug can interact with a particular protein. To further accelerate process and reduce cost, drug–target affinity has been explored in some studies. Drug–target affinity is a key property that determines the strength of the interaction between the small molecule drug and the target. The commonly used datasets for predicting drug–target affinity are the Kinase (Davis et al., 2011) and KIBA (Tang et al., 2014) datasets.

(2) Feature extraction and processing. Accurate and comprehensive descriptions of the biological or chemical functional information of drugs and targets in numerical form play an important role in the construction of high-performance models. Feature extraction of drugs and targets can be performed from different perspectives (Cheng, 2019; Zhao T. et al., 2020). For example, iGPCR-Drug (Xiao et al., 2013) obtains drug features by discrete Fourier transform of drug molecular fingerprints and extracts GPCR features according to pseudo amino acid compositions. DrugE-Rank (Yuan et al., 2016) represents drug features according to general descriptors and extracts target features according to amino acid composition, transformation, and distribution. TargetGDrug (Hu J. et al., 2016) extracts drug features by applying wavelet transform to drug molecular fingerprints and extracts GPCR features according to evolutionary information. Ru et al. (2020) extracted protein features using the distance-based top-n-gram algorithm and obtained drug features according to general descriptors. Chemical databases store information in a textual representation and the simplified molecular input line entry specification (SMILES) format is a common standard used in many cheminformatics software. Each SMILES string encodes structural information that can be used to predict complex chemical properties, and a large number of machine learning models can extract molecular features of compounds according to SMILES strings. Recently, convolutional neural networks (CNNs) and recurrent neural networks have been used for molecular feature extraction. Hirohara et al. (2018) transformed SMILES strings into two-dimensional matrices and used CNNs to extract molecular features. Goh et al. (2017) applied natural language processing to SMILES feature extraction and used recurrent neural networks for molecular strings.

The presence of invalid or redundant features not only reduces the accuracy of the experiment result but also lengthens the experimental period. Low-dimensional and comprehensive information feature sets are expected. Therefore, a variety of methods for processing features have been applied to related rearch (Zou et al., 2016a, b; Guo et al., 2020; Zhang G. et al., 2020; Zhao X. et al., 2020). For example, to reduce the noise between features, Li et al. (2017) used principal component analysis (PCA) to reduce the dimensionality of drugs and targets features. Tabei et al. (2012) combined 881 substructures of drugs and 876 Pfam domain structures of targets by tensor product to form feature vectors of drug–target pairs. MFDR (Hu P.-W. et al., 2016) used autoencoders as the building blocks of a deep network to reconstruct drug and protein features into a low-dimensional new representation. DeepConv-DT (Lee et al., 2019) used CNNs on raw protein sequences to capture local amino acid residue information by convolving amino acid subsequences of various lengths.

(3) Selection of task algorithms. Several task algorithms have been used for drug–target interaction prediction, such as classification algorithms, learning to rank algorithms, and deep learning algorithms (Cheng et al., 2019; Lv et al., 2019; Tao et al., 2020; Zhang Y. et al., 2020).

Most of the existing studies treat drug–target interaction prediction as binary tasks, and different classification algorithms have been applied. For example, Bleakley and Yamanishi (2009) proposed a bipartite local model (BLM) based on a support vector machine (SVM) kernel to predict drug–target relationships. LRF-DTI (Shi et al., 2019) is a drug–target interaction prediction method using Lasso for feature extraction and random forest for classification. Yamanishi et al. (2010) used a distance learning algorithm as a classifier. Pred-binding (Shar et al., 2016) extracted molecular structure and protein sequence features, and used support vector machines and random forests to classify whether drugs and targets can be docked.

Drug–target interaction prediction can be regarded as a ranking task. Exploring the strength of drug–target interactions can shorten the drug development process and save expenses. Zhang et al. (2015) applied six learning to rank algorithms (Prank, RankNet, RankBoost, SVMRank, AdaRank, and ListNet) to virtual screening of drugs, their study showed that learning to rank is an effective computational strategy, especially because of its novel use in cross-target virtual screening and heterogeneous data integration. DrugE-Rank (Yuan et al., 2016) used protein amino acid composition, transformation and distribution information, compound descriptor information, and output information of six classifiers as features to be input into the learning to ranking algorithm to improve the performance of drug-target interaction prediction.

Neural networks have also been used to solve related problems in the prediction of drug–target interactions. Prado-Prado et al. (2011) used the entropy information of drug–protein complexes and neural networks to predict drug–target affinity values. DeepDTA (Öztürk et al., 2018) proposed a deep-learning based model that used only sequence information of both targets and drugs, One novel approach used in this work is the modeling of protein sequences and compound 1D representations with CNNs. GraphDTA (Nguyen et al., 2019) focused on the fact that molecules are by nature formed by chemical bonding of atoms, and used graph convolutional network to learn drug-target binding affinity.

## Discussion

Under the background of the existing chemical and biological computing theory, big data and rapid development of computer technology, the use of machine learning for drug-target interaction prediction does have many benefits, but there are still some problems that need to be further explored.

(1) Data heterogeneity. Most of the existing studies are based on publicly available data in databases that collect data with different focuses, and each database has its own criteria for judging the data. Drugs, targets, and related data from different databases often have different terminological descriptions and different organization structures, such inconsistencies make data integration difficult.

(2) Effective representation of biological and chemical features. Feature engineering is a key concern in building machine learning models. There are often technical difficulties in how to effectively extract key features and how to deal with data with high dimensionality. Existing studies have shown that the features of proteins and drugs can be extracted from a variety of angles, and the combination of information from these angles can achieve complementary effects. Most drug–target interaction prediction studies only extract relatively one-sided information, and do not comprehensively consider the information from multiple perspectives. In addition, most studies have focused on extracting drug molecule and protein features separately, ignoring the potentially valid association that may exist between drug and target. Moreover, the direct concatenation of biologically unrelated features may lead to a decrease in prediction accuracy.

(3) Characteristics of task algorithms. The classification, ranking, or deep learning methods used in drug–target interaction prediction all have their own characteristics. Different computational approaches can be used to solve different problems in drug–target interaction prediction, however, these algorithms also have shortcomings. Classification is the simplest and most understandable task. However, there is an obvious and long-standing defect in this task that it is necessary to collect negative samples. Most existing classification studies take experimentally validated drug–target pairs with known interactions as positive samples, and unvalidated or unknown drug–target pairs as negative examples. Among these negative examples, there may be positive samples that have not been accurately validated, the performance of a model that is based on such a dataset will be biased.

On the basis of the existence of one-to-many or many-to-many relationships between queries and documents, learning to rank can be used in multi-target drug discovery. Early drug development followed the “one drug, one target” principle, with the aim of finding high-affinity, high-selective drugs for a specific receptor associated with a particular disease. However, the number of complex diseases is increasing and the proteins associated with these diseases are not limited to one, therefore drug combinations are used to achieve the optimal therapeutic effect. Clinical pharmacology studies have shown that drug combinations greatly increase the incidence of adverse drug reactions, but because of the lack of multi-target drugs, such risks have to be taken. Multi-target drugs are undoubtedly an important area for future research. Therefore, using the characteristics of learning to rank to tackle the multi-target problem of drugs deserves to be explored further. Learning to rank was originally applied for information retrieval. Its output is a relative score of correlation between queries and documents (Cheng, 2020; Ru et al., 2021). This is not sufficient for studies that require accurate prediction of drug–target affinities.

The use of neural networks for predicting accurate drug–target affinity values has shown great potential in this research area. Neural networks can fuse drug and target features, which have changed the current situation of simple concatenation or tensor products of drug and target features. Deep learning contains more neural network structures with multiple implicit layers compared with traditional machine learning, which allows deep learning to handle large datasets and identify complex patterns from the learning process. But for the same reason, neural networks require much more execution time than classification or ranking algorithms. It will lead to overfitting when the drug and target feature dimensions are high.

Although existing machine learning methods have opened a new area in drug–target interaction prediction, they have not achieved satisfactory results so far. Therefore, there is still a need to develop new theoretical and computational methods for drug–target interaction prediction.

## Conclusion

Drug–target interaction prediction can help to screen out unsuitable compounds and is an important step in the development of new drugs. In this review, we describe the importance of drug–target interaction prediction, analyze in detail the three main reasons why machine learning has become a mainstream technique, summarize the specific applications of machine learning methods in each step of building machine learning models, analyze the shortcomings of existing research methods, and discuss several aspects that can be further explored (Wei et al., 2014, 2017a, 2017b, 2018, 2019; Ding et al., 2017, 2019, 2020a, 2020b; Jin Q. et al., 2019; Jin S. et al., 2019; Li J. et al., 2020; Su et al., 2020; Wang H. et al., 2020; Zeng et al., 2020c, d; Zhai et al., 2020; Wang et al., 2021b). This review provides meaningful perspectives for future drug–target interaction prediction studies, especially the application of learning to rank to deal with multi-target drug problems.

## Author Contributions

XR drafted the manuscript. LX and RS initiated the idea, conceived the whole process, and finalized the manuscript. All authors have read and approved the final manuscript.

## Conflict of Interest

The authors declare that the research was conducted in the absence of any commercial or financial relationships that could be construed as a potential conflict of interest. The handling editor declared a past co-authorship with one of the authors, LX.

## Footnotes

1 https://www.uniprot.org/

2 https://pubchem.ncbi.nlm.nih.gov/

3 https://go.drugbank.com/

4 https://www.genome.jp/kegg/

5 https://www.bindingdb.org/bind/index.jsp

6 http://stitch.embl.de/

7 http://www.swisstargetprediction.ch/

8 https://www.rdkit.org/

9 https://mariewelt.github.io/OpenChem/html/index.html

10 https://ifeature.erc.monash.edu/

11 http://bioinformatics.hitsz.edu.cn/Pse-in-One/
